# Analysis of guide wire displacement in robot-assisted spinal pedicle screw implantation

**DOI:** 10.1007/s11701-024-01876-z

**Published:** 2024-03-30

**Authors:** Qing Liu, RuiYang Wang, Neng Ru, Yu Wu, ChangJin Guo, LeYuan Chen, Jie Liang, Fan Zhang

**Affiliations:** Orthopedics Department, The First College of Clinical Medical Science, China Three Gorges University, Yichang, China

**Keywords:** Robot-assisted surgery, Guide wire displacement, Pedicle screw insertion

## Abstract

Robot-assisted pedicle screw placement is prone to guide wire migration, and the related influencing factors have not yet been discussed. Therefore, this study aimed to investigate and analyze the causes of robot-assisted spinal pedicle guide wire displacement and summarize the relevant treatment strategies. The surgical outcomes of 82 patients who underwent robotic-assisted pedicle screw spinal placement at our hospital between July 2022 and June 2023 were retrospectively analyzed. A total of 342 screws were placed in 82 patients; 47 guide wires were offset, 47 guide wires were replaced, and 295 guide wires were not significantly offset, with a first guide wire offset rate of 13.7% and a total guide wire offset rate of 12.1%. Univariate analysis showed that Screw placement level, whether respiration was controlled during guide wire placement, Hu value of CT, the position of needle insertion point, and operation time had a significant effect on guide wire deviation (*P* < 0.05). Multivariate logistic regression analysis showed that the inclusion of screw placement segments, whether breathing was controlled during guide wire placement, and Hu value of CT had a significant effect on guide wire offset (*P* < 0.05). Whether the guide wire was offset had no significant effect on the accuracy of subsequent pedicle screw implantation (*P* > 0.05). The level of screw placement, whether breathing was controlled during guide wire placement, and Hu value of CT were independent risk factors for guide wire deviation. When causing an excursion, screw orientation can be adjusted during intraoperative screw placement, and guide wire excursion has no significant impact on the accuracy of subsequent pedicle screw placement.

## Introduction

In recent years, with the rapid development of artificial intelligence and the digital medical field, its application in the medical field has gradually become extensive. Robot navigation technology has significantly improved the excellent rate of pedicle screw placement due to its stable and accurate characteristics, showing excellent safety and accuracy. Robot-assisted screw placement has been reported to be excellent in 94.5–98.7% [1–5]. The excellent rate of free-hand pedicle screw placement ranged from 25.0 to 93.5% [2, 5, 6]. The accuracy of screw placement varies significantly between physicians, and the accuracy of screw placement is closely related to the physician's proficiency. Robot-assisted screw placement shortens the learning curve of young physicians and reduces the horizontal gap in pedicle screw placement between young and senior physicians, and it has taken surgical homogenization to a new level. At present, the accuracy and safety of robot-assisted screw placement have been widely recognized, but it is still found during the operation that the guide wire deviation during screw placement leads to screw deviation, especially in the learning stage. This study aimed to analyze the causes of robot-assisted spinal pedicle guide wire displacement, avoid guide wire displacement during surgery as much as possible, further improve screw placement accuracy, and reduce surgical complications.

## Subjects and methods

Patients who underwent spinal robot-assisted pedicle screw placement at our hospital between July 2022 and June 2023 were included. All the selected cases were patients with thoracolumbar fractures. The operator is from the same surgical team. The surgical robot was a TiRobot II orthopedic surgical robot. The composition of the TiRobot II system: a flexible robot arm, an optical tracking system, and a surgical planning workstation (TINAVI Medical Technology Co., Ltd.)

### Inclusion criteria

(1) Patients who underwent robotic-assisted pedicle screw fixation in our hospital due to thoracolumbar fractures and had screws with a diameter of 6.5 mm;

(2) No obvious surgical contraindications;

(3) Patients who underwent intraoperative C-arm 3D scanning after intraoperative guide wire placement;

(4) Patients with complete preoperative and postoperative imaging data and intraoperative experimental data records.

### Exclusion criteria

(1) Patients with previous spinal surgery;

(2) Patients with imperfect records of image data and experimental data;

(3) Patients who changed their surgical approach during surgery.

### Surgical technique

**Fig. 1:**
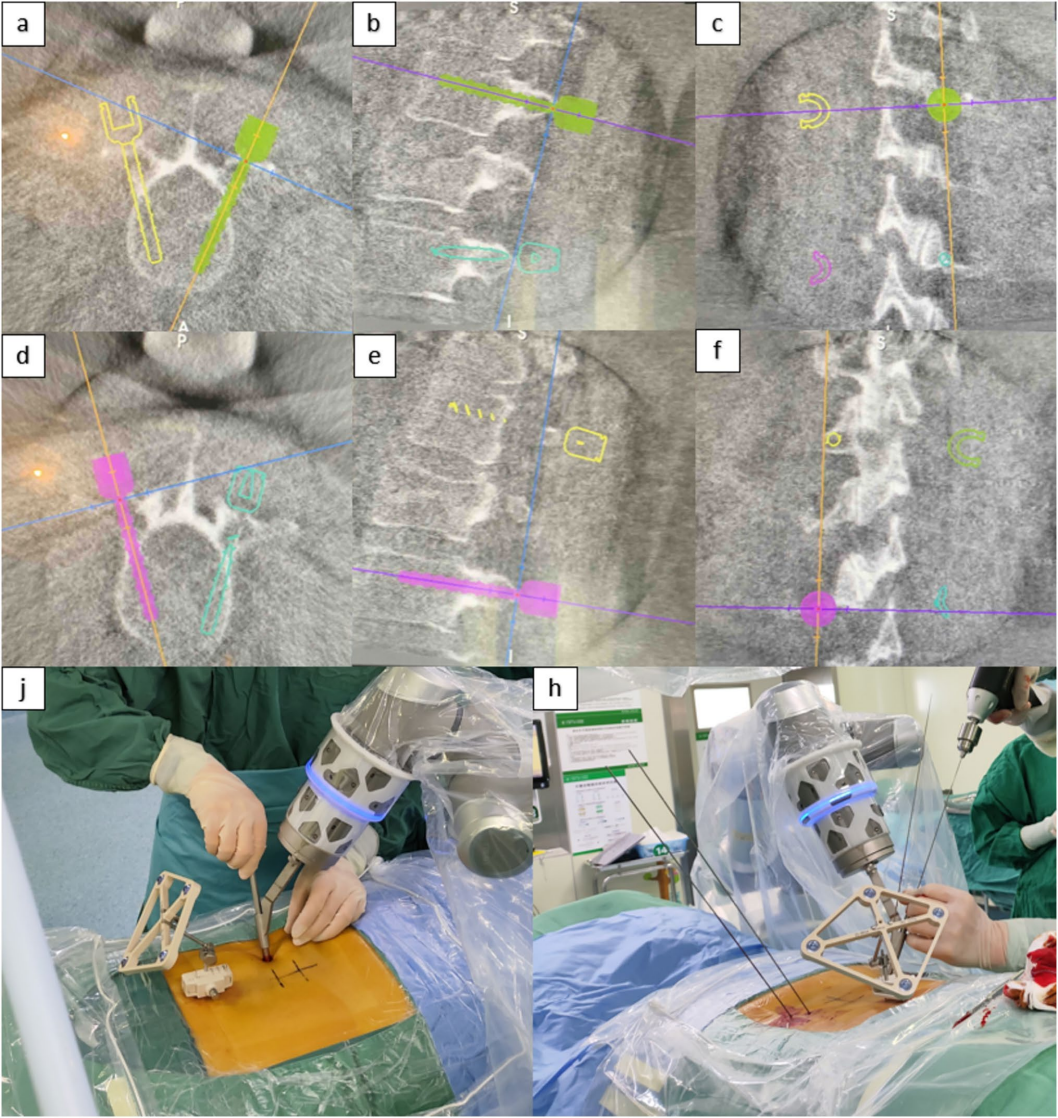
**a**–**f** Planning process of guide wire and screw trajectory in robot computer; **j**–**h** Incision of skin and soft tissue and placement of guide sleeve, and insertion of guide wire along the sleeve

**Fig. 2:**
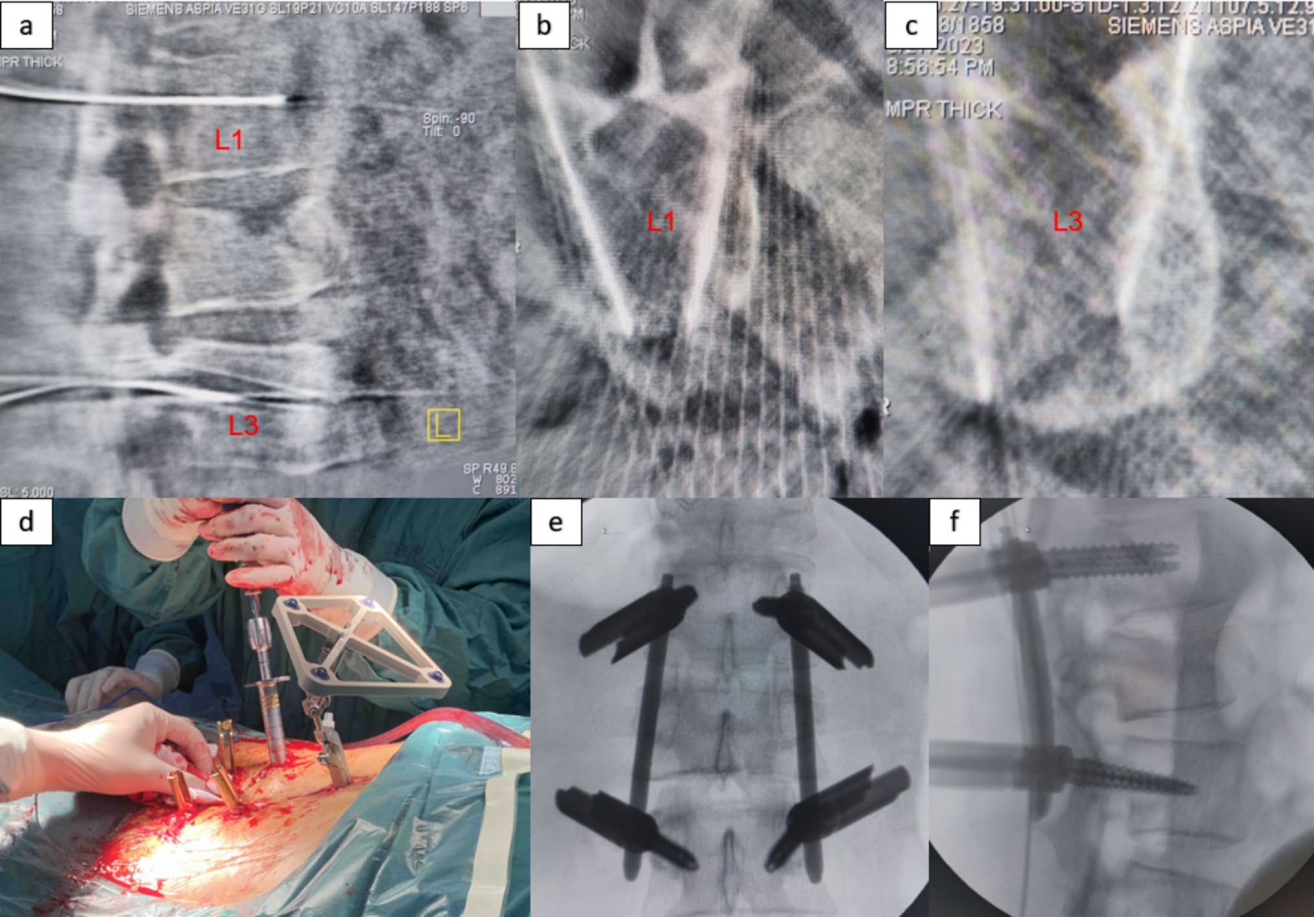
**a** Intraoperative 3D scan sagittal view; **b** Guide wire position in L1 vertebral body; **c** Guide wire position in L3 vertebral body; **d** Pedicle screw placement over a guide wire; **e** and **f** fluoroscopic images after screw placement

Patients were placed in the prone position after successful anesthesia. Routine disinfection and draping of the surgical area. Fix the positioner adjacent to the spinous process or posterior superior iliac spine of the patient's surgical incision (it is appropriate not to interfere with the robotic arm operation of the robot). Place the registration device in the surgical field as close to the skin as possible. The target vertebral body was scanned by C-arm 3D, and the data were synchronized to the surgical robot computer. The operator performs navigation planning based on 3D images of the patient's vertebral body (Fig. 1a–f). After planning is completed, the robotic arm automatically places the screw guide in the needle insertion path. Insert the sleeve into the screw guide. Surgeons cut skin, fascia, and muscle tissue along the sleeve at the point where the sleeve contacts the skin. Continue to advance the sleeve down the incision to the bone surface. The registration information was determined again, and the guide wire was placed into the vertebral body through the sleeve after successful registration (Fig. 1j–h). According to the above operation, place the guide wire through the pedicle at the target vertebral body according to the robot's planning path in turn. An intraoperative 3D scan was performed after the guide wire placement to confirm whether the guide wire position coincided with the preoperative planning (Fig. 2a–c). If the guide wire position is found to be unsatisfactory, reposition the guide wire following the appealing steps. After confirming the satisfactory position of the guide wire, the soft tissue is expanded step-by-step along the guide wire with an expansion sleeve. Cortical opening and tapping were performed at the insertion point along the direction of the guide wire, and then pedicle screws with a diameter of 6.5 mm were placed along the guide wire (Fig. 2d–f).

### Diagnostic criteria for guide wire displacement

**Fig. 3:**
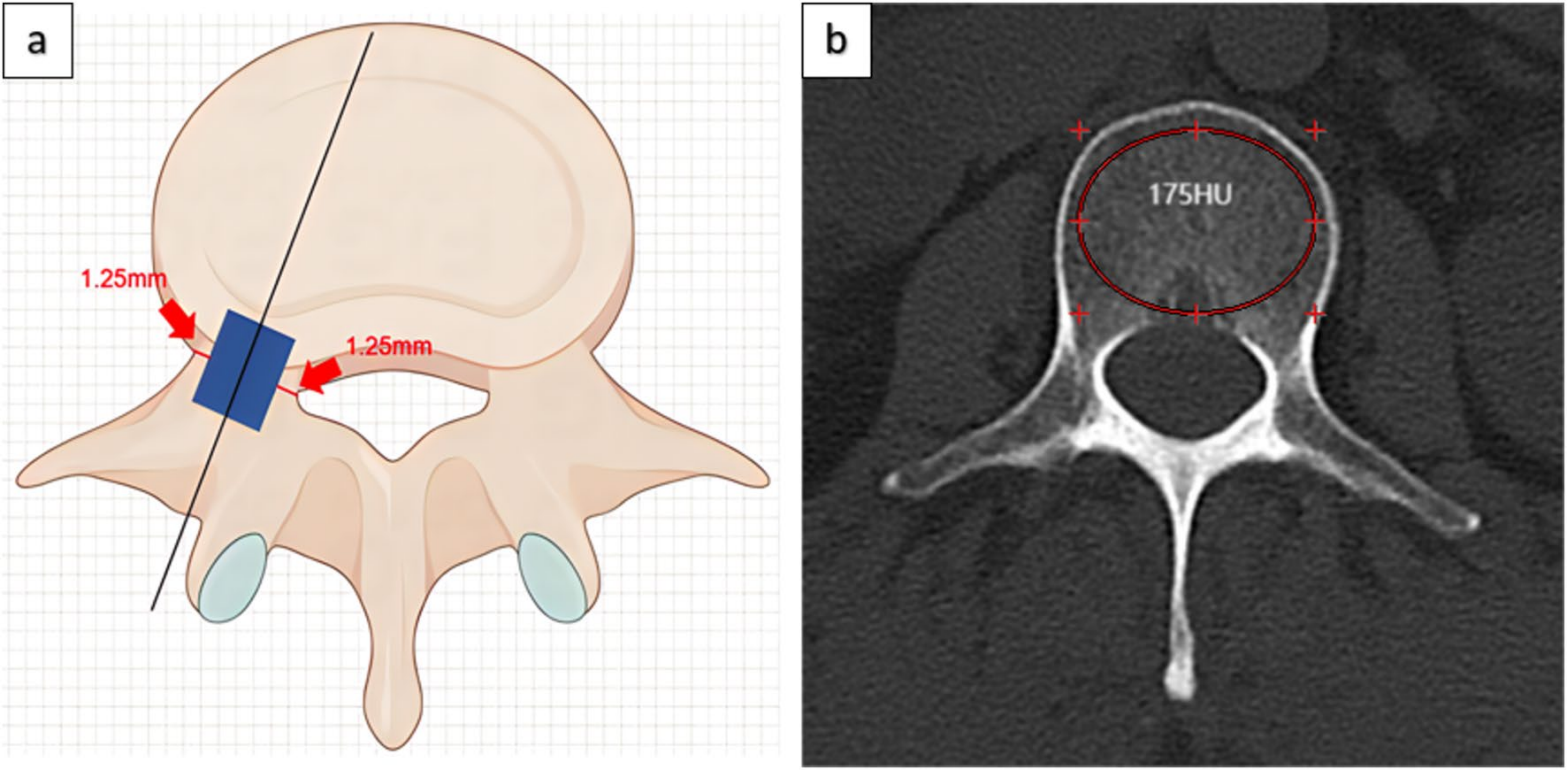
**a** Grade A guide wire in blue area of pedicle and Grade B guide wire outside blue area; **b** Hu value of vertebral body

In this study, 6.5 mm diameter cannulated pedicle screws were used during surgery. According to Gertzbein-Robbins accuracy grading criteria for pedicle screws [7], we attempted to grade guide wire accuracy in this study (Fig. 3a):

Grade A: The guide wire is completely within the pedicle, and the shortest distance from the pedicle wall is > 1.25 mm (corresponding to Gertzbein-Robbins grades A and B).

Grade B: distance from guide wire to pedicle wall ≤ 1.25 mm, or outside the pedicle (corresponding to Gertzbein-Robbins grades C–E).

Grade A is defined as no significant guide wire deviation (acceptable range), and Grade B is defined as significant guide wire deviation (unacceptable range). For grade B guide wier, we will replan the route to the correct position.

### Observation index

(1) Sociodemographic factors such as sex, age, BMI, smoking history, drinking history, and nature of work;

(2) Number of screws placed during operation, operation time, type of surgical incision (transverse incision is perpendicular to the longitudinal axis of the body, vertical incision is parallel to the longitudinal axis of the body), Control respiration during guide wire placement (control breath time of 60 to 180 s), needle insertion point, Hu value of vertebral body CT (The mean Hu value in the region of interest (ROI) was automatically measured by the PACS system through the mid-vertebral body level of the transverse vertebral body image of the CT-positioned screw, and the ROI was selected in the mid-vertebral body level plane to avoid cortical bone and posterior venous plexus regions [8]) (Fig. 3b). Intraoperative C-arm 3D scans were performed to assess whether the guide wire was significantly offset.

### Statistical analysis

All data were analyzed using SPSS 23.0 software. The normal distribution of measurement data was expressed as the mean ± standard deviation (*x̅* ± s), the skewed distribution was expressed as the median and interquartile range, and the enumeration data were expressed as frequency and percentage. Independent sample *t* test or rank sum test was used for comparison between groups, and Chi-square test was used for comparison of enumeration data. *P* < 0.05 was considered statistically significant. Logistic regression analysis was performed for factors that were statistically significant in univariate analysis. *P* < 0.05 was considered statistically significant, and *P* < 0.01 was considered highly significant.

## Results

### Characteristic of patients

A total of 82 patients were included in this study, including 48 males and 34 females. The mean patient age was 52 years, with an age range of 24–63 years. BMI ranged from 17.10 to 34.48 kg/m^2^, with a mean of 22.78 kg/m^2^. The average operation time was 76.5 min. A total of 342 screws were placed, with an average screw placement of about 4 screws per person (Table 1).

**Table 1:** Clinical characteristics of patients with robotic screw placement

Variables		Overall (*N* = 82)
Age(years)		52 (48,57)
Gender	Male	48 (58.5%)
	Female	34 (41.5%)
BMI(kg/m^2^)		22.78 (20.76,24.92)
Smoking history		38 (46.3%)
Drinking history		37 (45.1%)
Number of screws	4	76 (92.7%)
	6	5 (6.1%)
	8	1 (1.2%)
Operation time(min)		76.50 (65.75,96.25)
Occupation	Farmers	33 (40.2%)
	Retired personnel	6 (7.3%)
	Staff	10 (12.2%)
	Worker	22 (26.8%)
	Freelancers	6 (7.3%)
	Unemployed	4 (4.9%)
	Student	1 (1.2%)
Guide wire displacement	No	66 (80.5%)
	Yes	16 (19.5%)

### Univariate analysis of risk factors for guide wire displacement

The patients were divided into two groups: the group with an obvious deviation from the guide wire and the group without an obvious deviation. The analysis showed that gender, age, BMI, smoking history, drinking history, number of nails placed, and nature of work had no significant effect on guide wire deviation (*P* > 0.05). There was a statistical difference in operation time between the two groups (*P* < 0.05) (Table 2).

**Table 2:** Univariate analysis of risk factors for guide wire displacement

Risk factors		No displacement	Displacement	Correlation coefficient	*P*
Age(years)		52 (47.75, 52.25)	52 (49.25, 57.75)	− 0.616	0.538
Gender	Male	39 (59.1%)	9 (56.3%)	0.043	0.836
	Female	27 (40.9%)	7 (43.8%)		
BMI(kg/m^2^)		22.91 (20.71, 25.70)	22.49 (21.48, 23.95)	− 0.398	0.691
Smoking history	Yes	30 (45.5%)	8 (50.0%)	0.107	0.744
	No	36 (54.5%)	8 (50.0%)		
Drinking history	Yes	30 (45.5%)	7 (43.8%)	0.015	0.902
	No	36 (54.5%)	9 (56.3%)		
Number of screws	4	63 (95.5%)	13 (81.3%)	5.109	0.085
	6	3 (4.5%)	2 (12.5%)		
	8	0 (0.0%)	1 (6.3%)		
Occupation	Farmers	27 (40.9%)	6 (37.5%)	2.111	0.965
	Retired personnel	5 (7.6%)	1 (6.3%)		
	Staff	8 (12.1%)	2 (12.5%)		
	Worker	16 (24.2%)	6 (37.5%)		
	Freelancers	5 (7.6%)	1 (6.3%)		
	Unemployed	4 (6.1%)	0 (0.0%)		
	Student	1 (1.5%)	0 (0.0%)		

A total of 342 screws were placed on 82 patients. Guide wires were divided into groups according to whether there was a significant deviation of guide wires during screw placement, including 47 cases of guide wire deviation, 47 times of reinsertion of guide wires, and 295 cases of guide wires without significant deviation. The guide wire's first offset rate was 13.7%. The overall guide wire offset rate was 12.1%. Univariate analysis showed that the deviation of guide wire was significantly affected by the level of screw placement, whether the respiration was controlled during the placement of guide wire, Hu value of CT, and the position of needle insertion point (*P* < 0.05). The type of surgical incision and guide wire offset had no significant effect (*P* > 0.05) (Table 3).

**Table 3:** Univariate analysis of risk factors for guide wire displacement

Risk factors		No displacement	Displacement	Correlation coefficient	*P*
Type of incision	Transverse incision	129(43.7%)	15(31.9%)	2.321	0.128
	Vertical incision	166(56.3%)	32(68.1%)		
Level of screw placement	Thoracic vertebra	156(52.9%)	16(34.0%)	5.755	0.016
	Lumbar vertebra	139(47.1%)	31(66.0%)		
Control respiration	Yes	249(84.4%)	29(61.7%)	13.739	0.000
	No	46(15.6%)	18(38.3%)		
Hu value		160.13 ± 44.32	174.83 ± 34.01	− 2.629	0.010
Position of needle insertion point	Superior part of accessory process	12(4.1%)	6(12.8%)	6.152	0.013
	Base of accessory process	283(95.9%)	41(87.2%)		

### Multivariate analysis of risk factors for guide wire displacement

Multivariate logistic regression equations were constructed by including the screw placement level, whether breathing was controlled during guide wire placement, Hu value of CT, and needle insertion point. Results showed that uncontrolled breathing increases the risk of guide wire offset, which is statistically significant (OR = 0.252, 95%CI 0.111–0.570, *P* = 0.001). Statistically significant higher risk of guide wire offset during lumbar screw placement compared to thoracic (OR = 0.470, 95%CI 0.241–0.915, *P* = 0.026). The higher the Hu value of CT, the higher the risk of guide wire deviation during screw placement, which is statistically significant (OR = 0.988, 95%CI 0.980–0.996, *P* = 0.004). Point of insertion has no significant effect on guide wire offset (*P* > 0.05) (Table 4).

**Table 4:** Multivariate analysis of risk factors for guide wire offset during screw placement

Risk factors		*β*	SE	Wald	OR	95% CI for Exp (B)		*P*
						Lower part	Upper part	
Control respiration	Yes※							
	No	− 1.380	0.417	10.951	0.252	0.111	0.570	0.001
Level of screw placement	Thoracic vertebra※							
	Lumbar vertebra	− 0.756	0.340	4.937	0.470	0.241	0.915	0.026
Hu value		− 0.012	0.004	8.283	0.988	0.980	0.996	0.004
Position of needle insertion point	Superior part of accessory process※							
	Base of accessory process	− 0.279	0.618	0.203	0.757	0.255	2.543	0.652

※: Control group

**Fig. 4:**
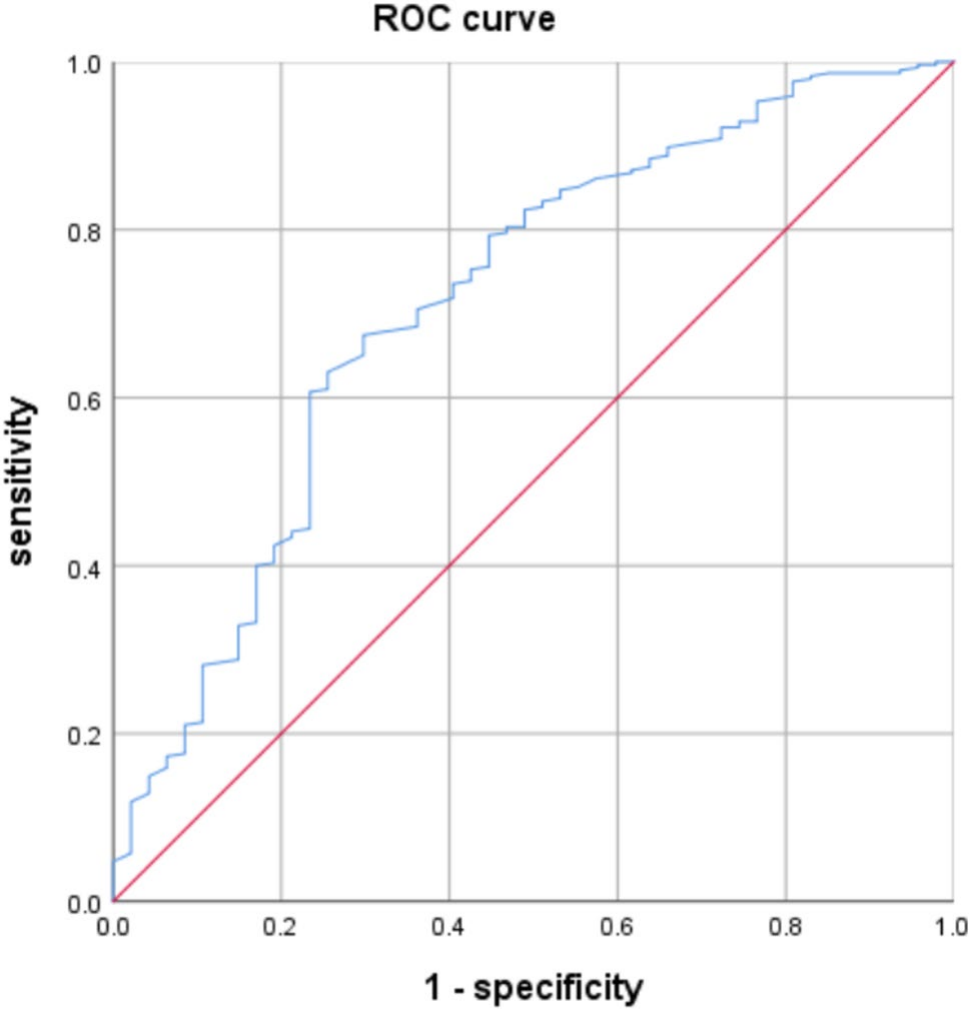
ROC curve analysis of prediction model for guide wire offset during screw placement

The multivariate analysis yielded significant factors, including predictors such as “level of screw placement, control respiration, and Hu value of CT,” which were integrated to establish a logistic regression equation: *P* = 1/(1 + e-Y), e is the base of the natural logarithm, Y = (− 1.380 × Control respiration) + ( − 0.756 × level of screw placement) + ( − 0.012 × Hu value of CT)—5.432. Predicted values PRE-1 were automatically generated for this model, with newly generated PRE-1 as the test variable, and whether the guide wire is offset or not is a state variable. Receiver operating characteristic (ROC) curves were plotted to evaluate the discriminatory power of the model. AUROC = 0.714,95% CI: 0.630–0.798. The goodness of fit test *P* = 0.761 > 0.05 by the Hosmer–Lemeshow test shows that there is no statistical difference between the current model and the perfect model in the ideal, and the goodness of fit is good (Fig. 4).

### The relationship between guide wire displacement and pedicle screw implantation

The analysis showed that a total of 342 screws were placed, including 300 grade A screws, 40 grade B screws, and 2 grade C screws, and whether the guide wire was offset had no significant effect on the accuracy of subsequent pedicle screw implantation (*P* > 0.05) (Table 5).

**Table 5:** Relationship between guide wire offset and operation time and screw implantation

		No displacement	Displacement	Correlation coefficient	*P*
Pedicle Screw Grade	A	259(87.7%)	41(87.2%)	0.344	0.857
	B	34(11.5%)	6(12.8%)		
	C	2(0.7%)	0(0.0%)		

### Learning curve of robot-assisted screw placement in the spine

**Fig. 5:**
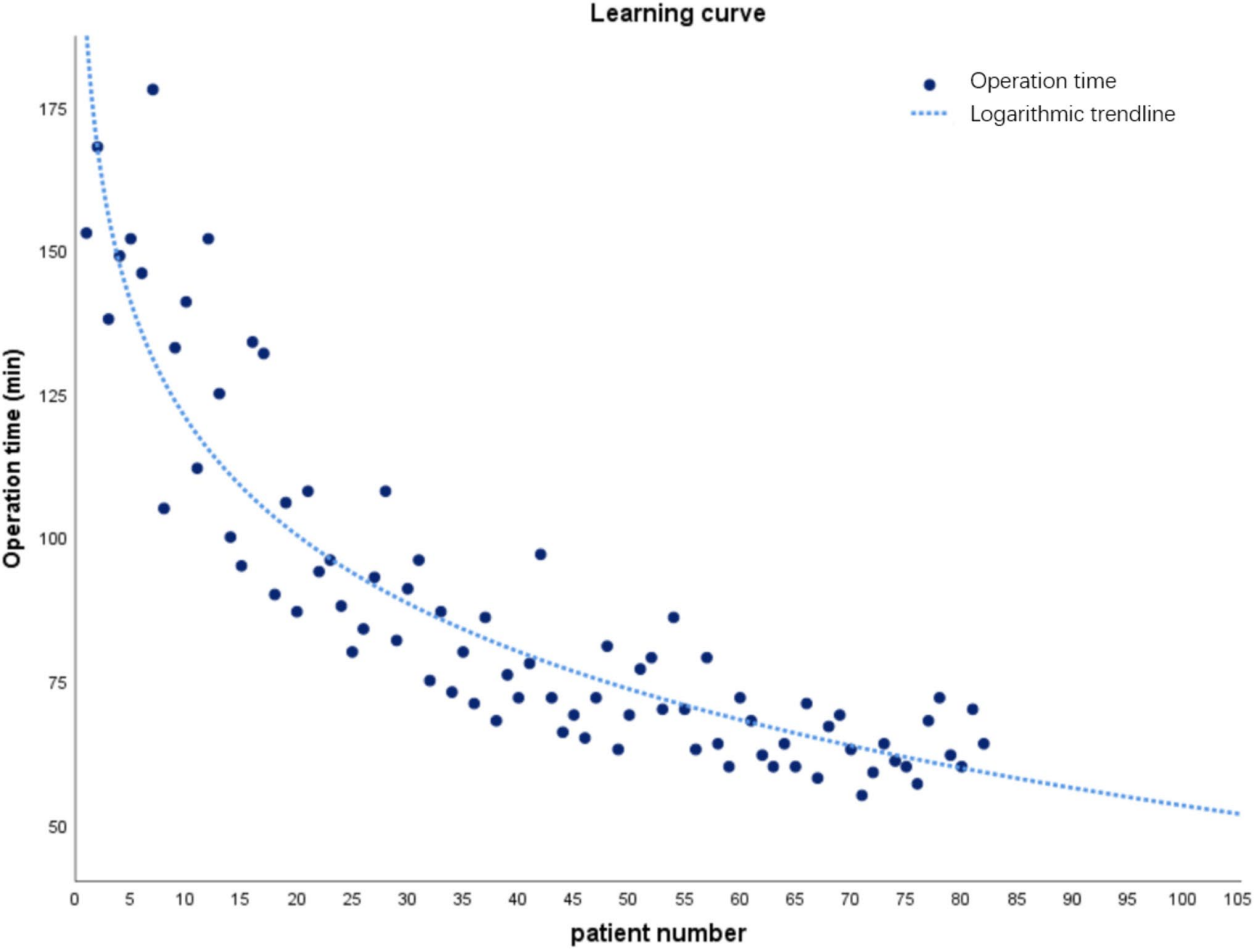
Curve fitting using logarithmic regression between operative time and number of operation cases

Robot-assisted spinal pedicle screw implantation learning curve was assessed using regression analysis and a scatter plot with logarithmic curve fitting. The fitting equation was y = aln(x) + b. x was the patient number and y was the operation time. A scatter plot might show how the number of patients affects the pattern of operational time fluctuations. The results showed that the average operation time of 82 patients was 87.20 min, the shortest time was 55 min, and the longest time was 178 min. Through logarithmic curve fitting regression analysis, we found that the operative time (*y* = − 29.285ln[x] + 188.075, *R*^2^ = 0.827; *p* < 0.001) significantly decreased as operation cases increased. The operation time was greatly reduced after 20 cases, and the curve began to stabilize (Fig. 5).

## Discussion

At present, the mainstream of pedicle screw technology is free-hand operation. The free-hand screw placement technique has the advantages of short time, relative accuracy, and small radiation, but its accuracy depends heavily on the surgeon’s technique and experience. There are significant differences in the accuracy of screw placement among different physicians, and the excellent rate of screw placement among senior physicians is difficult to replicate. Complications associated with pedicle screw placement are common, and the incidence of neuro-related complications of our greatest concern is 0.37–1% [9, 10]. There has been rapid development in the field of spinal robotics in the past 10 years. Spinal robots have obvious advantages in operation stability, accuracy, replicability, and safety, which significantly improve the excellent and good rate of pedicle screw placement, and also reduce the technical threshold and shorten the learning curve [11]. However, a significant deviation in the guide wire and pedicle screws was still found in our study.

The TiRobot II orthopedic surgical robot used in this study belongs to the navigation robot. It constructs three-dimensional data of the target vertebral body by intraoperative 3D scanning to completely match the relative position of the vertebral body to the body surface localizer. Then, the navigation system detects the relative positions of the robot localizer and patient body surface localizer and guides the robot to find the target vertebral body and planning path. In this study, the guide wire perforated the lateral wall of the pedicle in three cases and the medial wall in five cases, but did not perforate the superior and inferior walls of the pedicle. Postoperative reexamination showed that pedicle screws were grade C in two cases. After pedicle screw placement, fluoroscopy was performed in the anteroposterior and lateral lumbar spine, and screw invasion into the spinal canal was suspected in one case, and manual modification was performed. In one patient, the guide wire perforated the medial pedicle cortex, and the patient developed numbness in the lower extremities after surgery, which disappeared after 2 weeks of conservative treatment. The remaining patients had no complications.

Obesity has previously been found to be an independent risk factor for robot-assisted screw offset [11, 12]. During the operation, we realized that the difficulty of the operation and the number of excursions in obese patients were significantly higher than those in normal patients. Because the guide wire offset rate was higher due to the lack of experience in the early stages of this experiment, it is possible that the statistical results could not reflect the effect of BMI on guide wire offset. According to multivariate regression analysis, the level of screw placement, whether respiration was controlled during guide wire placement, and Hu value of CT were independent risk factors for guide wire deviation. The lumbar spine has a more hypertrophic muscle mass relative to the thoracic spine, has a longer surgical channel, and is more easily pulled by soft tissue during guide wire placement to change the predetermined needle insertion trajectory. With the improvement of operation proficiency, the difference in thoracic and lumbar guide wire deviation rates gradually decreased in the later experiment. We believe that the impact of this factor may no longer be meaningful when operational techniques are further improved and the sample size is sufficiently large. Because the patient is in the prone position, the patient's breathing amplitude is too large during 3D scanning with CT during surgery, resulting in errors in the relative position of the vertebral body to the localizer or matching failure [6, 13]. This causes errors in the planning of the needle insertion path, leading to guide wire offset. The Hu value of vertebral body CT reflects bone quality, and it is more accurate and convenient than bone mineral density measurement [14–16]. In this study, it was found that the larger Hu value of vertebral body CT was more likely to lead to guide wire deviation. According to the literature, there is no relevant report at present. We believe that when the guide wire is not rigid enough and resistance at the current end is high, the operator will apply more force to the guide wire, and the guide wire may advance toward an area with relatively little resistance around it causing the guide wire to shift the predetermined path.

**Fig. 6:**
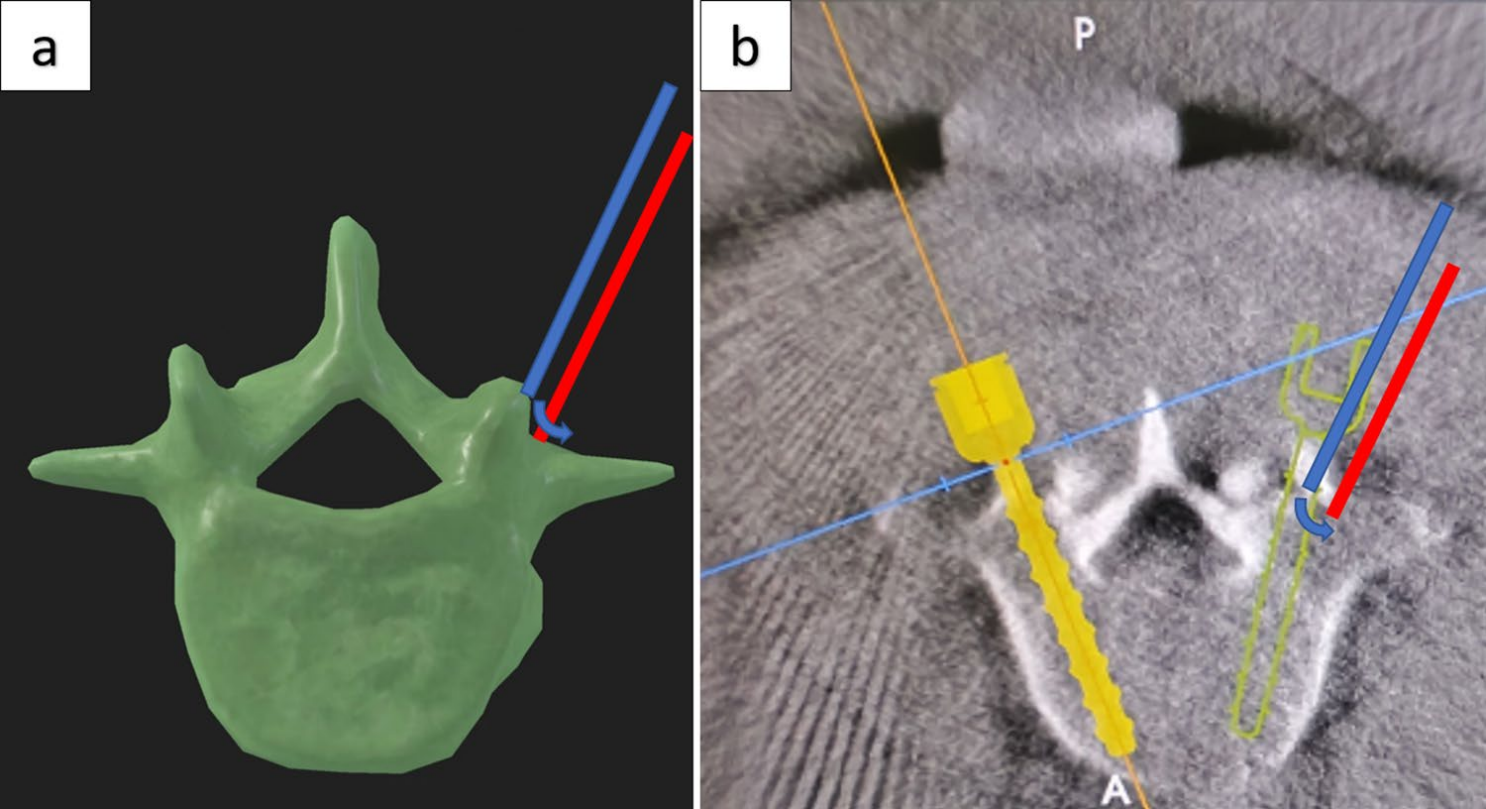
The blue and red lines in the figure represent the guide sleeve. **a** Diagram showing sleeve sliding principle on bone surface; **b** Intraoperative planning diagram showing sleeve sliding principle on bone surface

In the univariate analysis of this study, when the needle insertion point was at the base lateral to the accessory process, the guide wire offset rate was significantly lower than that of the needle insertion point on the accessory process. The choice of insertion point for preoperative planning is usually near the accessory process, which is a ridge extending outwardly and inferiorly from the superior facet. When the insertion point is selected in the accessory process, slippage is more likely to occur when the sleeve reaches the bone surface due to the steep bone surface at this site [2, 12, 13, 17, 18]. While the bone surface at the base of the accessory process is relatively flat, the sleeve is more stable here (Fig. 6). However, there was no significant statistical difference in this factor in multivariate regression analysis, indicating that needle insertion point selection at the base lateral to the accessory process or accessory process was not an independent risk factor for guide wire deviation. The influencing factors of needle insertion point were not considered in 12 operations before our experiment. In the 13th case, we selected the insertion point at the base lateral to the accessory process to try to avoid slippage of the sleeve on the bone surface. The first 12 patients had only 18 needle insertion points during surgery, and we chose to be in the accessory process. Because the sample size is too small, it may lead to biased statistical results. The influence of this factor on guide wire offset is also closely related to the operator's operation.

We found that whether the guide wire was offset had no significant effect on the accuracy of subsequent pedicle screw placement. We performed a CT scan after guide wire placement during surgery, and the guide wire with a significant deviation would be corrected in time. We observed that the excellent and good rate of screws was only 99.3% and not 100%. There were two Grade C screws. The CT values of the vertebral bodies in which these two screws were located were 92 HU and 44 HU, respectively, suggesting severe osteoporosis in these two vertebral bodies. During free-hand screw placement, some degree of screw swing inevitably occurs, and the swing phenomenon may lead to screw erosion around the pedicle [19]. In severely osteoporotic vertebral bodies, it is easy for guide wire screws to deviate in a predetermined direction or even perforate the pedicle cortex resulting in nerve injury.

According to the analysis of operation time, we concluded that the learning curve of the robot-assisted nailing technique was about 20 cases, and this result was consistent with that reported by other scholars [20–23]. However, some studies have also reported no difference in learning curve between experienced physicians and residents for robot-assisted screw placement techniques [11]. We still recommend having at least one experienced senior physician on the surgical team to ensure that the procedure is performed safely.

### Modified method

1. Selection of incision: It is recommended to incise the skin along the sleeve direction to reach the bone surface when establishing the surgical channel. Minimize distraction from the sleeve by the surrounding soft tissue. Try to select a vertical incision. Although the choice of transverse incision and vertical incision had no significant effect on guide wire deviation, according to this experiment, the transverse incision caused increased intraoperative blood loss and more significant postoperative incision pain.

2. When planning the needle insertion point, the lateral base of the accessory process was selected as much as possible. This reduces the chance of sleeve slippage. When advancing the sleeve to the bone surface, gently touch the bone surface to avoid slippage of the sleeve on the bone surface as a result of advancing the sleeve.

3. When the first 3D scan obtains the data from the surgical vertebral body, if the patient has good oxygen saturation, the mechanical ventilation can be temporarily turned off (60–180 s) to avoid matching errors.

4. CT scans are recommended after robot-assisted placement of the guide wire to check the accuracy of the guide wire. The anteroposterior and lateral views of the intraoperative X-rays do not truly reflect the guide wire position information.

### Limitations

This study has some limitations. First, the patient has to undergo at least 2 3D scans in a single procedure (there may be cases where a 3D scan fails, intraoperative image matching fails, etc., requiring re-scanning). This significantly increases the radiation exposure of the patient. We did not find other accurate, easy, and less side-effect methods of assessing guide wire accuracy instead of intraoperative 3D scans. Second, the lack of clarity of intraoperative 3D scanning images may lead to deviations in the accuracy of intraoperative measurement guidewires, thus affecting the experimental results. Third, this study was a single-center retrospective study with a small sample size. Finally, the surgical team in this study has experienced chief physician members who will learn robotic experience from other surgical teams during this period. This may cause errors in learning curve studies of robotics.

## Conclusion

The level of screw placement, whether breathing was controlled during guide wire placement, and Hu value of CT were independent risk factors for guide wire deviation. When causing an excursion, screw orientation can be adjusted during intraoperative screw placement, and guide wire excursion has no significant impact on the accuracy of subsequent pedicle screw placement. According to the learning curve analysis, the learning curve of the robot-assisted pedicle screw placement technique was about 20 cases.

## Data Availability

The datasets used and/or analyzed during the current study are available from the corresponding author on reasonable request.

## References

[1] Volk VL, Steele KA, Cinello-Smith M, Chua RV, Pollina J, Poulter G, Shafa E, Busselberg P, Fitzpatrick CK (2023) Pedicle screw placement accuracy in robot-assisted spinal fusion in a multicenter study. Ann Biomed Eng 51(11):2518–2527. 10.1007/s10439-023-03291-1 37458895 10.1007/s10439-023-03291-1 PMC12090060

[2] Han X, Tian W, Liu Y, Liu B, He D, Sun Y, Han X, Fan M, Zhao J, Xu Y et al (2019) Safety and accuracy of robot-assisted versus fluoroscopy-assisted pedicle screw insertion in thoracolumbar spinal surgery: a prospective randomized controlled trial. J Neurosurg Spine. 10.3171/2018.10.SPINE18487 30738398 10.3171/2018.10.SPINE18487

[3] Kantelhardt SR, Martinez R, Baerwinkel S, Burger R, Giese A, Rohde V (2011) Perioperative course and accuracy of screw positioning in conventional, open robotic-guided and percutaneous robotic-guided, pedicle screw placement. Euro Spine J: Off Publ European Spine Soc, Euro Spinal Deformity Soc, Euro Sect Cervical Spine Res Soc 20(6):860–868. 10.1007/s00586-011-1729-2 10.1007/s00586-011-1729-2 PMC3099153 21384205

[4] Devito DP, Kaplan L, Dietl R, Pfeiffer M, Horne D, Silberstein B, Hardenbrook M, Kiriyanthan G, Barzilay Y, Bruskin A et al (2010) Clinical acceptance and accuracy assessment of spinal implants guided with SpineAssist surgical robot: retrospective study. Spine 35(24):2109–2115. 10.1097/BRS.0b013e3181d323ab 21079498 10.1097/BRS.0b013e3181d323ab

[5] Yan K, Zhang Q, Tian W (2022) Comparison of accuracy and safety between second-generation TiRobot-assisted and free-hand thoracolumbar pedicle screw placement. BMC Surg 22(1):275. 10.1186/s12893-022-01723-8 35840958 10.1186/s12893-022-01723-8 PMC9288055

[6] Tian W, Liu YJ, Liu B, He D, Wu JY, Han XG, Zhao JW, Fan MX (2019) Guideline for thoracolumbar pedicle screw placement assisted by orthopaedic surgical robot. Orthop Surg 11(2):153–159. 10.1111/os.12453 31025807 10.1111/os.12453 PMC6594520

[7] Gertzbein SD, Robbins SE (1990) Accuracy of pedicular screw placement in vivo. Spine 15(1):11–14. 10.1097/00007632-199001000-00004 2326693 10.1097/00007632-199001000-00004

[8] Zaidi Q, Danisa OA, Cheng W (2019) Measurement techniques and utility of hounsfield unit values for assessment of bone quality prior to spinal instrumentation: a review of current literature. Spine 44(4):E239-e244. 10.1097/BRS.0000000000002813 30063528 10.1097/BRS.0000000000002813

[9] Gautschi OP, Schatlo B, Schaller K, Tessitore E (2011) Clinically relevant complications related to pedicle screw placement in thoracolumbar surgery and their management: a literature review of 35,630 pedicle screws. Neurosurg Focus 31(4):E8. 10.3171/2011.7.FOCUS11168 21961871 10.3171/2011.7.FOCUS11168

[10] Wang Y, Kahaer A, Maimaiti A, Guo H, Rexiti P (2023) Complication, fusion, and revision rate in the lumbar cortical bone trajectory and pedicle screw fixation techniques: a systematic review and meta-analysis. J Orthop Surg Res 18(1):382. 10.1186/s13018-023-03820-7 37226223 10.1186/s13018-023-03820-7 PMC10210483

[11] Torii Y, Ueno J, Iinuma M, Yoshida A, Niki H, Akazawa T (2023) Accuracy of robotic-assisted pedicle screw placement comparing junior surgeons with expert surgeons: can junior surgeons place pedicle screws as accurately as expert surgeons? J Orthop Sci: Off J Jpn Orthop Assoc 28(5):961–965. 10.1016/j.jos.2022.06.012 10.1016/j.jos.2022.06.012 35864030

[12] Zhang JN, Fan Y, Hao DJ (2019) Risk factors for robot-assisted spinal pedicle screw malposition. Sci Rep 9(1):3025. 10.1038/s41598-019-40057-z 30816334 10.1038/s41598-019-40057-z PMC6395613

[13] Du W, Zou D, Zhang J, Liu J, Qu W, Zhang S (2021) Guide wire displacement in robot-assisted spinal pedicle screw implantation. Wideochirurgia i inne techniki maloinwazyjne = Videosurgery and other miniinvasive techniques 16(3):526–535. 10.5114/wiitm.2021.103952 34691302 10.5114/wiitm.2021.103952 PMC8512515

[14] Buenger F, Sakr Y, Eckardt N, Senft C, Schwarz F (2022) Correlation of quantitative computed tomography derived bone density values with Hounsfield units of a contrast medium computed tomography in 98 thoraco-lumbar vertebral bodies. Arch Orthop Trauma Surg 142(11):3335–3340. 10.1007/s00402-021-04184-5 34562119 10.1007/s00402-021-04184-5 PMC9522714

[15] Di M, Weng Y, Wang G, Bian H, Qi H, Wu H, Chen C, Dou Y, Wang Z, Ma X et al (2023) Cortical endplate bone density measured by novel phantomless quantitative computed tomography may predict cage subsidence more conveniently and accurately. Orthop Surg 15(12):3126–3135. 10.1111/os.13897 37853959 10.1111/os.13897 PMC10694013

[16] Chen L, Wu XY, Jin Q, Chen GY, Ma X (2023) The correlation between osteoporotic vertebrae fracture risk and bone mineral density measured by quantitative computed tomography and dual energy X-ray absorptiometry: a systematic review and meta-analysis. Euro Spine J: Off Publ Euro Spine Soc, Euro Spinal Deformity Soc, Euro Sect Cervical Spine Res Soc 32(11):3875–3884. 10.1007/s00586-023-07917-9 10.1007/s00586-023-07917-9 37740786

[17] Molliqaj G, Schatlo B, Alaid A, Solomiichuk V, Rohde V, Schaller K, Tessitore E (2017) Accuracy of robot-guided versus freehand fluoroscopy-assisted pedicle screw insertion in thoracolumbar spinal surgery. Neurosurg Focus 42(5):E14. 10.3171/2017.3.FOCUS179 28463623 10.3171/2017.3.FOCUS179

[18] Schatlo B, Molliqaj G, Cuvinciuc V, Kotowski M, Schaller K, Tessitore E (2014) Safety and accuracy of robot-assisted versus fluoroscopy-guided pedicle screw insertion for degenerative diseases of the lumbar spine: a matched cohort comparison. J Neurosurg Spine 20(6):636–643. 10.3171/2014.3.SPINE13714 24725180 10.3171/2014.3.SPINE13714

[19] Faldini C, Viroli G, Fiore M, Barile F, Manzetti M, Di Martino A, Ruffilli A (2021) Power-assisted pedicle screws placement: Is it as safe and as effective as manual technique? Narrative review of the literature and our technique. Musculoskelet Surg 105(2):117–123. 10.1007/s12306-021-00714-x 34050490 10.1007/s12306-021-00714-x PMC8324584

[20] Torii Y, Ueno J, Iinuma M, Yoshida A, Niki H, Akazawa T (2022) The learning curve of robotic-assisted pedicle screw placements using the cumulative sum analysis: a study of the first 50 cases at a single center. Spine Surg Related Res 6(6):589–595. 10.22603/ssrr.2022-0049 10.22603/ssrr.2022-0049 PMC9747205 36561165

[21] Akazawa T, Torii Y, Ueno J, Umehara T, Iinuma M, Yoshida A, Tomochika K, Ohtori S, Niki H (2023) Learning curves for robotic-assisted spine surgery: an analysis of the time taken for screw insertion, robot setting, registration, and fluoroscopy. Euro J Orthop SurgTraumatol: Orthop Traumatologie. 10.1007/s00590-023-03630-x 10.1007/s00590-023-03630-x 37358731

[22] Chen X, Song Q, Wang K, Chen Z, Han Y, Shen H, Li Q (2021) Robot-assisted minimally invasive transforaminal lumbar interbody fusion versus open transforaminal lumbar interbody fusion: a retrospective matched-control analysis for clinical and quality-of-life outcomes. J Comp Eff Res 10(10):845–856. 10.2217/cer-2021-0078 33906371 10.2217/cer-2021-0078

[23] Lee NJ, Lombardi JM, Boddapati V, Mathew J, Leung E (2021) Clinical and patient-reported outcomes after robot-assisted short-segment lumbar fusion with a minimum 1-year follow-up. Interdiscip Neurosurg 25:7. 10.1016/j.inat.2021.101168

